# Exploring the Microbiome’s Impact on Glioma and Brain Metastases: Insights into Development, Progression, and Treatment Response—A Scoping Review

**DOI:** 10.3390/cancers17071228

**Published:** 2025-04-04

**Authors:** Jennifer Leigh, Becky Skidmore, Adrian Wong, Saman Maleki Vareki, Terry L. Ng

**Affiliations:** 1 Division of Medical Oncology, Department of Medicine, The Ottawa Hospital Cancer Centre, Ottawa, ON K1Y 4E9, Canada; jleigh@toh.ca; 2 Mount Sinai Hospital, Toronto, ON M5G 1X5, Canada; 3 Skidmore Research & Information Consulting Inc., Ottawa, ON, Canada; becky.skidmore.rls@gmail.com; 4 Faculty of Medicine, University of Ottawa, Ottawa, ON K1H 8M5, Canada; adrian.wong@uottawa.ca; 5 Department of Pathology and Laboratory Medicine, Western University, London, ON N6A 3K7, Canada; saman.malekivareki@lhsc.on.ca; 6 Verspeeten Family Cancer Centre, London Health Sciences Research Institute, London, ON N6A 5W9, Canada; 7 Department of Oncology, Western University, London, ON N6A 3K7, Canada; 8 Department of Medical Biophysics, Western University, London, ON N6A 3K7, Canada

**Keywords:** microbiome, brain tumor, dysbiosis, glioma, glioblastoma, brain–gut axis

## Abstract

**Background**: The human microbiome plays a crucial role in health and disease. Dysbiosis, an imbalance of microorganisms, has been implicated in cancer development and treatment response, including in primary brain tumors and brain metastases, through interactions mediated by the gut–brain axis. This scoping review synthesizes current evidence on the relationship between the human microbiome and brain tumors. **Methods**: A systematic search of five electronic databases was conducted by an expert librarian, using controlled vocabulary and keywords. A targeted grey literature search in Google Scholar and clinical trial registries was also undertaken. Eligible studies included primary research involving human patients, or in vivo, or in vitro models of glioma or brain metastasis, with a focus on the microbiome’s role in tumor development, treatment response, and outcomes. **Results**: Out of 584 citations screened, 40 studies met inclusion criteria, comprising 24 articles and 16 conference abstracts. These included 12 human studies, 16 using mouse models, 7 combining both, and 5 employing large datasets or next-generation sequencing of tumor samples. Thirty-one studies focused on primary brain tumors, six on brain metastases, and three on both. Of the 20 studies examining dysbiosis in tumor development, 95% (n = 19) found an association with tumor growth. Additionally, 71.4% (n = 5/7) of studies reported that microbiome alterations influenced treatment efficacy. **Conclusions**: Although the role of the gut–brain axis in brain tumors is still emerging and is characterized by heterogeneity across studies, existing evidence consistently supports a relationship between the gut microbiome and both brain tumor development and treatment outcomes.

## Simple Summary

The relationship between the microbiome and cancer pathogenesis has been previously well documented for some types of solid tumors, but its impact on brain tumor development is less clear. This review sought to highlight the current existing literature surrounding the interaction between the microbiome, primary and metastatic brain tumor development, and their response to treatment. We identify a robust association between the microbiome and brain tumor development, with emerging data supporting a bidirectional relationship where the microbiome may predict treatment response and cancer therapies impact the host microbiome. A lot of the information, however, comes from preclinical studies, and more clinical studies are needed to better understand this relationship.

## 1. Introduction

The human microbiome, consisting of 500–1000 bacterial species and numerous fungal and viral species, coexists within the body and is shaped by diet, environment, medications, and genetics [1,2]. The microbiome composition varies widely among individuals and across the lifespan. Although no definitive consensus exists on what constitutes a ‘healthy’ microbiome, dysbiosis has been linked to numerous diseases, with certain bacterial species identified as pathogenic [1]. A growing area of research explores the microbiome’s role in cancer development, progression, and treatment response.

The relationship between the microbiome and cancer pathogenesis has been well documented, with gut dysbiosis implicated in the development of colorectal, biliary tract, oral, and gynecologic cancers [3,4]. Notable examples include *Helicobacter pylori*-induced gastritis, leading to gastric cancer, and *Schistosoma haematobium*, contributing to bladder cancer [4,5]. This influence is largely mediated through immune system modulation, with the gut microbiota influencing both innate and adaptive immune responses. Mechanisms include the activation of CD8+ T cells and T helper 1 cells, which contribute to antitumor immunity [6,7].

The microbiome also influences cancer treatment outcomes, primarily through immune modulation. In melanoma, the efficacy of cytotoxic T-lymphocyte associated protein 4 (CTLA-4) blockade has been associated with an increased abundance of *Bacteroides thetaiotaomicron* and *Bacteroides fragilis*, which enhance Th1-mediated immune responses [8]. Similarly, *Bifidobacteria* species improve T cell priming and response to programmed death-ligand 1 (PD-L1) blockade in pre-clinical melanoma models [9]. Beyond immunotherapy, the microbiome influences other treatments; for example, *Akkermansia muciniphila* enhances the efficacy of abiraterone acetate in prostate cancer by exerting anti-inflammatory effects [10].

Despite significant advances in understanding the microbiome’s role in extracranial cancers, its relationship with brain tumors—both primary and metastatic—remains underexplored. Brain metastases are more common than primary brain tumors, with gliomas being the most prevalent primary brain malignancy [11]. Glioblastoma multiforme (GBM), the most aggressive glioma subtype, has a median survival of only 15–18 months despite multimodal treatment [12]. In contrast, the prognosis of patients with brain metastasis varies depending on the primary tumor site. While the mechanisms by which the microbiome influences brain tumors remain poorly understood, proposed pathways include reduced neurotransmitter receptor expression, bacterial metabolite production altering immune responses, and increased blood–brain barrier permeability, which facilitates immune suppression and cancer cell immune escape [13,14,15].

The gut–brain axis describes the bidirectional communication between the central nervous system (CNS) and the enteric nervous system, with growing evidence suggesting that this axis mediates interactions between the microbiome and brain tumors. Glioma mouse models have shown that tumor development induces gut microbiome alterations, mirroring changes observed in human fecal studies of patients inflicted by glioma [14]. Certain bacterial species are more prevalent in the gut microbiome of patients with isocitrate dehydrogenase (IDH)-wild type glioma and in the oral microbiome of patients of those with high-grade gliomas compared to low-grade gliomas [16]. However, these findings are based on small case series or in vitro studies, with no consensus on how the microbiome interacts with brain tumor development and treatment response.

This scoping review aims to summarize the current evidence on the microbiome’s role in brain tumors, focusing on its relationship with tumor development, progression, and treatment response in both primary and metastatic brain tumors. By consolidating and analyzing existing data, this review seeks to clarify the uncertainties surrounding the gut–brain axis and its implications for brain tumor research and treatment.

## 2. Methods

### 2.1. Protocol

This scoping review was written in accordance with the preferred reporting items for systematic reviews and meta-analyses extension for scoping reviews (PRISMA-Scr) guidance [17].

### 2.2. Eligibility Criteria

Studies were included in this scoping review if they met the following criteria: (1) involved human, animal, or in vitro models of glioma (including oligodendroglioma, diffuse astrocytoma, oligoastrocytoma, anaplastic oligodendroglioma, anaplastic astrocytoma, glioblastoma) or brain metastasis; (2) focused on the relationship between oral and gut microbiome compositions and glioma or brain metastasis development, response to both systemic and local therapies, and/or overall outcomes; and (3) were primary research studies with the following designs: randomized control trials, cohort studies, case–control studies, or case report with more than five patients. Full texts and conference abstracts were included. All clinical, in vivo and in vitro studies were included, with no restrictions on country of origin or publication year. Additionally, the references of relevant review articles were screened to ensure a comprehensive inclusion of studies.

### 2.3. Information Sources and Search

An experienced medical information specialist (IS) developed the search strategy through an iterative process in consultation with the review team. Another senior IS peer reviewed the MEDLINE strategy prior to execution with the PRESS Checklist [18]. Using the Ovid platform and applying the multifile option and deduplication tool available, we searched Ovid MEDLINE^®^ ALL, Embase Classic+Embase, EBM Reviews—Cochrane Central Register of Controlled Trials, and EBM Reviews—Cochrane Database of Systematic Reviews. We also searched the Web of Science (core databases). The initial search was conducted on 6 January 2023, and it was updated on 8 May 2024 (Appendix A—Search Strategy).

The searches incorporated a combination of controlled vocabulary (e.g., “Glioma”, “Microbiome”, “Dysbiosis”) and keywords (e.g., “brain tumor”, “gut flora”, “brain–gut interplay”), and vocabulary and syntax were adjusted as necessary across the databases. There were no language, date, or population restrictions on any of the searches. We downloaded and deduplicated the database results using EndNote 9.3.3 (Clarivate Analytics) and subsequently uploaded them to Covidence (Veritas Health Innovation Ltd., Melbourne, Australia).

### 2.4. Study Selection

**Figure 1:**
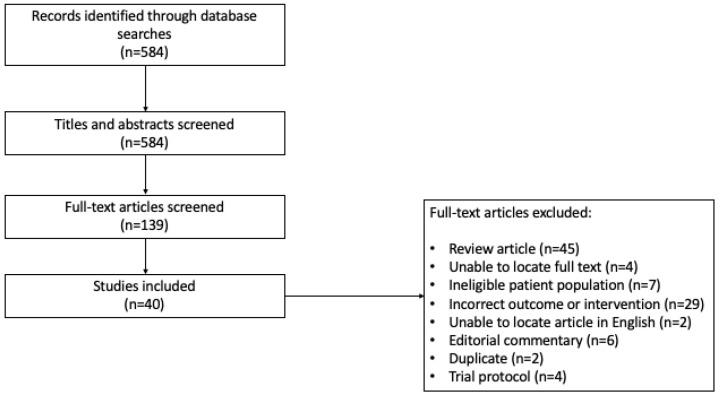
Search strategy for studies included in the scoping review (Prisma Flow Diagram).

Level I screening of title and abstracts and level II screening of full-text articles and abstracts were completed independently by two reviewers based on the eligibility criteria (Figure 1). Study selection was not blinded. Any conflict was discussed between the two reviewers, and a consensus was reached. We performed a grey literature search in Google Scholar, ClinicalTrials.gov, and the ICTRP Search Portal.

### 2.5. Extraction of Data

Data were independently extracted by the primary reviewer using a standardized data collection form in Microsoft Excel. The following information was systematically recorded for each study: primary author, location, journal, publication year, type of publication, study design, sample source, microbiome detection method, population (human, animal, in vitro), type of brain tumor, antibiotic or probiotic receipt, systemic therapy receipt, type of systemic therapy received, brain radiotherapy receipt, whether or not surgery occurred, and microbiome signatures associated with glioma or brain metastasis presence, growth, and treatment response. Missing or unpublished data were also documented.

### 2.6. Outcome Measures

The primary objective of this scoping review was to summarize the existing evidence on the interaction between the microbiome and brain tumors. Secondary objectives included identifying microbiome signatures associated with glioma presence and growth, systemic therapy responses, and radiotherapy responses. These objectives were examined across human, animal, and in vitro studies.

## 3. Results

### 3.1. Characteristics of Selected Studies

The initial and updated search resulted in 584 citations; 139 articles after stage I (title and abstract) review, and then 40 studies deemed eligible for data extraction after stage II (full text) review (Figure 1). Of these, 24 were full-text articles and 16 were abstracts. Twelve studies included human subjects only, sixteen utilized mouse models only, seven included both, and five studies used machine learning from large datasets or next-generation sequencing on tumor samples. A total of 1462 patients were included in the human studies, of which 1010 (69.1%) had a brain cancer diagnosis, and 452 (30.9%) were healthy controls; one study, available as an abstract only, did not provide the sample size. In total, there were 737 patients (73.0%) with primary brain tumors and 273 (27.0%) with brain metastases.

Of the 40 eligible studies, 31 studies focused on primary brain tumors, 6 on brain metastases, and 3 on both primary and metastatic brain tumors. The majority of studies (n = 29) examined the gut microbiome using fecal samples, with other microbiome sources including oral (n = 5), tumor (n = 3), and serum (n = 1). Two studies examined tumor growth dynamics without direct stool microbiome measurement by depleting the gut microbiome with antibiotics. Most studies were conducted in China (n = 17, 42.5%) or the United States of America (n = 15, 37.5%) and were published in 2021 or later (n = 34, 85.0%). A summary of study characteristics is presented in Table 1

### 3.2. Study Characteristics: Microbiome and Brain Tumor Development Relationship

A total of 29 studies examined microbiome changes associated with primary (n = 24) and secondary (n = 7) brain tumors, including 14 mouse studies, 16 human studies, and 3 using large genome datasets (Table 2) [16,19,20,21,22,23,24,25,26,27,28,29,30,31,32,33,34,35,36,37,38,39,40,41,42,43,44]. Microbiome signatures were primarily determined using 16S ribosomal ribonucleic acid (rRNA) sequencing (n = 19), followed by 16S ribosomal deoxyribonucleic acid (rDNA) sequencing (n = 5), and shotgun metagenomic sequencing (n = 4). One additional study reported using 16S sequencing but did not specify whether it used RNA or DNA. Additionally, seven studies examined metabolomic changes, five using liquid chromatography-mass spectrometry, and two using gas chromatography-mass spectrometry.

Of the studies that examined microbiome changes, 20 focused on the changes in the microbiome at the time of brain tumor development, diagnosis, and/or at the time of tumor growth. The majority (n = 19/20, 95.0%) identified microbiome alterations with brain tumor development, while one study (5.0%) found no significant microbiome changes. Of note, the study with no changes examined viral microbiome (virome), whereas other studies investigated the bacterial microbiome [37].

The impact of gut microbiome depletion on brain tumor growth with antibiotic use was explored in five studies [19,21,27,36,45]. Two studies showed increased tumor growth, two demonstrated decreased tumor growth, and one found no change with antibiotic administration. All studies utilized mouse models. One study reporting decreased tumor growth suggested altered T cell activity due to antibiotic-induced gut microbiome depletion as a possible mechanism [27], while a study showing increased tumor growth linked the effect to enhanced tumor vasculogenesis following antibiotic treatment [36]. Brain tumor growth was measured using histology and volume calculation via imaging software in three studies (60.0%), in vivo optical imaging in one study (20.0%), and was not reported in the final study, which was an abstract only. Lastly, two studies examining the role of probiotics found beneficial effects on glioma outcomes [20,39].

### 3.3. Microbiome Signatures Associated with Primary Brain Tumor Growth

A total of 24 studies focused on primary brain tumor growth, primarily investigating gliomas, with 12 human studies, 14 mouse models of glioma, and three using human genome datasets. Two studies also examined benign brain tumors [25,32]. Of these, 15 studies (62.5%) found that microbiome dysbiosis was associated with brain tumor development [14,16,20,21,22,24,25,29,30,31,32,34,41,42,44]. Additionally, three studies demonstrated increased tumor growth following gut microbiome depletion using antibiotics, while one study found no change in tumor size with antibiotic treatment [16,19,21,36]. The abundance of the phylum *Bacillota* was altered in brain tumor growth, but its association with brain tumor growth was inconsistent across studies.

Two studies examined the role of probiotics in glioma growth. One study by Fan et al. administered a *Bifodobacterium* mixture to mice with glioma cells and found that it increased median overall survival (mOS) from 42 days to 52 days (*p* < 0.05). The second study by Wang L et al. tested different probiotic cocktails in glioma-injected mice and found that supplementation with *Bifidobacterium lactus* and *Lactiplantibacillus plantarum* decreased glioma growth, likely through alterations in the PI3K/AKT pathway [39].

One study explored the association between microbiome composition and glioma grade using salivary samples from patients with high-grade gliomas (HGG) and low-grade gliomas (LGG). This study identified specific bacterial associations with glioma grade, finding that the abundance of *Patescibacteria* decreased significantly with increasing glioma malignancy, from LGG to HGG, while the abundance of other major phyla (*Bacillota*, *Bacteroidetes*, *Proteobacteria*, *Actinobacteria*, *Fusobacteria*, and *Spirochaetota*) remained unchanged [41]. Additionally, this study found that the abundance of *Bacillota* was significantly lower in patients with IDH-1 mutated gliomas compared to IDH wild type.

Two studies explored the microbiome as a biomarker for glioma development. Li et al. identified a combination of six genera (*Bifidobacterium*, *Bacteroides*, *Lachnospira*, *Fusobacterium*, *Parasutterella*, and *Escherichia/Shigella*) as a potential biomarker to differentiate patients with brain tumors from healthy controls [32]. Yang et al. developed a diagnostic model using extracellular vesicles (EVs) released by microorganisms, detected in the peripheral blood, which could differentiate patients with brain tumors from healthy controls [42]. Finally, three studies utilized large human datasets from prior genome-wide association studies (GWAS) to determine causal relationships between gut microbiota and GBM using mendelian randomization. All three studies, which used the same GWAS meta-analysis conducted by the MiBioGen consortium, found the bacterial family *Ruminococcaceae* to be protective against GBM [23,40,43].

### 3.4. Microbiome Signatures Associated with Brain Metastasis

All seven studies examining the gut or oral microbiome in metastatic brain tumors reported dysbiosis compared to controls (Table 2) [26,27,33,38,42,45,46]. These studies included human participants, with three also utilizing mouse models. The primary disease sites were non-small cell lung cancer (NSCLC, n = 4), melanoma (n = 1), or unspecified (n = 2). Three studies found altered alpha-diversity (within-sample diversity) and beta-diversity (between-sample similarity) associated with brain metastasis development [26,33,42].

One study identified phyla-level changes in patients with metastatic brain tumors, with increased *Bacillota* (formerly *Firmicutes*) and decreased *Actinobacteria* and *Proteobacteria* [42]. Two related studies associated *Pseudomonas aeruginosa* with brain metastases; it was highly abundant in the sputum and feces of patients with NSCLC and brain metastases, but absent in NSCLC without brain metastases and healthy controls [33,38]. Notably, beta-diversity differences were observed in sputum samples but not fecal samples. Another study linked decreased fecal abundance of the genus *Blautia* with brain metastases in NSCLC [45]. Lastly, in a separate study, distinct bacterial signatures were identified in the stool, saliva and buccal samples from patients with metastatic brain tumors compared to primary brain tumors [46].

### 3.5. Microbiome Signatures Associated with Treatment Response

Thirteen studies examined microbiome interactions with cancer treatments, including primary brain tumors and one study on brain metastasis from melanoma (Table 3) [14,16,24,31,34,47,48,49,50,51,52,53,54]. Treatments included radiotherapy (n = 2), temozolomide (TMZ, n = 7), anti-PD-1 (n = 5), bevacizumab (n = 1) and Delta-24-RGDOX viroimmunotherapy (n = 2). Six studies evaluated microbiome changes due to treatment, six examined microbiome impacts on treatment response, and one addressed both.

Of the studies assessing microbiome impact on treatment response, five (71.4%) found associations between microbiome composition and treatment efficacy [48,49,51,55]. Dees et al. demonstrated that microbiome composition influenced anti-PD-1 response but not TMZ efficacy in humanized mouse models, with higher fecal levels of *Bacteroides cellulosilyticus* and *Eubacterium* species correlating with response [47]. Similarly, Kim et al. transplanted feces from patients with GBM or metastatic melanoma to the brain (MBM) into mice, and observed varied anti-PD-1 responses based on gut microbiome composition [53]. Ongoing analysis seeks to identify specific microbial differences between responders and non-responders.

One study on pediatric diffuse intrinsic pontine glioma (DIPG) found that higher fecal levels of *Eubacterium* species and *Synergistaceae* correlated with radiotherapy response, while *Flavobacteriaceae* and *Bacillales* were associated with disease progression [48]. In contrast, Ladomersky et al. reported antibiotic-induced gut microbiome depletion did not affect the efficacy of radiotherapy +/− anti-PD-1 in mice [50]. In another study, probiotics did not augment anti-PD-1 treatment responses in a GBM mouse model [54].

Of the study’s treatment effects on the microbiome, five (71.4%) included TMZ [14,16,24,31,52]. All observed microbiome changes with TMZ. Three studies noted gut dysbiosis was associated with glioma growth [16,24,31]. Dono et al. found no changes in fecal microbiome diversity post-chemoradiotherapy in patients with glioma but reported genus-level shifts in TMZ-treated mice [16]. Similarly, Li et al. observed microbiome alterations in glioma mice treated with TMZ, including increased *Verrucomicrobia* at seven days after treatment and reduced Bacillota-to-Bacteroidetes ratio post-treatment [31].

### 3.6. Impact of Dietary Changes on the Microbiome and Brain Tumors

Three studies investigated the effects of dietary modifications on the gut microbiome and brain tumor outcomes (Table 4), all using mouse models: two for GBM and one for glioma, type not specified. Kim J et al. found that introducing a high glucose drink (HGD) five weeks before tumor inoculation improved survival in mice compared to normal drinking water, but post-tumor inoculation HGD supplementation had no effect [54]. HGD supplementation increased *Desulfovibrionaceae* abundance regardless of tumor status, and supplementation with *Desulfovibrio vulgaris* in microbiome-depleted mice enhanced survival in glioma-bearing mice. McFarland B et al. showed that a ketogenic diet slightly increased survival in glioma-bearing mice, with long-term survivors exhibiting elevated gut *Faecalibaculum rodentium*, suggesting its potential as a probiotic [56]. Kim H et al. supplemented tryptophan into the diet of GBM-bearing mice, improving survival in a microbiota-dependent manner, although specific microbial changes were unavailable due to the study’s abstract-only status [29].

### 3.7. Microbiome and the Immune System

There were 13 studies that explored possible mechanisms for how the microbiome influences brain tumor growth and response to therapy [19,21,23,24,26,29,30,34,35,36,39,47,54]. The majority of these (n = 7) suggest this may be through influence on the immune system [19,24,29,34,35,47,53]. Dees K et al. created different humanized microbiome mice with fecal samples from five different human donors [55]. In the mouse line that responded to anti-PD-1 therapy, there was a significant increase in CD8+ and CD4+ T-cells producing IFN-γ, as well as in the CD8/Tregs ratio, which was not seen in the non-responder line. Hou X et al. found that IL-1β and TNF-α were increased in mice who responded to TMZ, suggesting potential reversal of immunosuppression caused by glioma [24]. In a study where mice were treated with a CD4+ depleting agent and RGDOX/indoximod, they exhibited lower gut microbial richness compared to mice with functional CD4+ cells [34]. Specifically, the depleted mice had a decrease in *Bifidobacterium* and *Lactobacillus*. Finally, two studies demonstrated that dietary changes resulted in more potent cytotoxic T cell response [29,54].

## 4. Discussion

This scoping review summarized 40 studies on the interplay between the microbiome and brain tumors, including tumor growth, development, and response to systemic and radiotherapies. Despite heterogeneity and the field’s early stage of development, evidence suggests significant crosstalk between the microbiome and brain tumors. Specifically, these studies demonstrate that dysbiosis is associated with growth of both primary and secondary brain tumors, with 95.0% of the studies examining this relationship (n = 19/20) making this conclusion. This relationship was also found to be bidirectional, with dysbiosis leading to increased tumor growth, and tumor growth also leading to dysbiosis. This is further supported by the studies demonstrating that antibiotic-induced microbiome depletion correlates with glioma growth, underscoring the microbiome’s importance in brain tumor outcomes.

This influence appears to at least in part take place through immune system modulation. A number of studies suggested that manipulation of the microbiome either through dietary changes or antibiotics can lead to increased cytotoxic T cell activity and ultimately enhanced anti-tumor immune response. Additionally, the gut microbiome was found to influence response of brain tumors to systemic therapies through immunomodulation, specifically for TMZ and anti-PDL-1 agents. This interplay between the microbiome, immune system, and cancer, has been previously demonstrated in other disease sites such as melanoma [8]. Traditionally, however, the brain has been felt to be an immunoprivileged organ and deemed a ‘cold’ tumor with limited efficacy from immunotherapy to date [13]. These results highlight that the microbiome can likely influence the brain tumor and immune system relationship, and thus disease outcomes, and should be an area of further focus in human studies.

In other cancers, specific bacterial species such as *Bacteroides fragilis* and polyketide synthetase positive *Escherichia coli*, *Streptococcus gallolyticus*, and *Morganella morganii* have been linked to tumorigenesis [57]. Our review identified potential microbiome signatures for brain tumors. *Ruminococcaceae * was protective against GBM in genome-wide studies [28,40,43], while *Bifidobacterium* was enriched in healthy controls but depleted in primary brain tumors; its dietary supplementation improved outcomes in mice [32,39]. *Pseudomonas aeruginosa* was elevated in the sputum of patients with NSCLC and brain metastases [33,38], and HGD supplementation increased *Desulfovibrionacea* abundance, correlating with improved GBM outcomes. Dietary supplementation with these protective species is an interesting area for future focus, especially to determine whether it can influence patient outcomes.

The *Bacillota* phylum was frequently implicated as a dysbiosis marker [58], though its role remains ambiguous, as there was no consensus among studies on whether an increase or decrease in abundance was associated with tumor growth. Most of the studies in our review did find that dysbiosis was associated with brain tumor development. Furthermore, dysbiosis prevention or reversal was observed with TMZ in three studies [14,16,24]. Similar findings implicating gut dysbiosis as a mechanism in other diseases such as multiple sclerosis, Alzheimer’s, and Parkinson’s underscore the relevance of the gut–brain axis [59].

Several studies explored the interplay between treatment and microbiome. Four examined immunotherapy responses in primary glioma models, and one included both glioma and brain metastasis models. Three demonstrated microbiome-dependent response variability, echoing findings in other cancers [60]. For example, fecal microbiota transplantation (FMT) from immunotherapy responders restored treatment efficacy in antibiotic-treated mice [60]. Promising results from a phase I trial combining FMT with plus nivolumab or pembrolizumab in metastatic melanoma highlight this approach’s potential [61]. Although it is not yet standard for primary brain tumors, it remains an area of interest for brain metastases.

Overall, this field is rapidly evolving, with most studies included in our review published between 2021 and 2024. Four active clinical trials examining the microbiome (three in primary brain tumors, one in metastatic brain tumors) registered on ClinicalTrails.gov further indicate growing interest. Advancing our understanding of the microbiome–brain tumor relationship will likely yield novel therapeutic strategies in the coming years.

### Strengths and Limitations

This scoping review has several limitations. A significant portion of the data is derived from in vivo mouse models, which despite the use of human feces to simulate the human microbiome, fail to account for factors such as diet, genetics, environment, and medication use. This limits the direct applicability of the findings to humans. Additionally, variability in the quality and specificity of microbiome reporting—ranging from overall diversity to phylum-, genus-, species-level abundance—hinders direct comparisons across studies. Lastly, several studies included were conference abstracts without corresponding peer-reviewed articles, offering limited data for extraction. Stronger evidence from human studies with standardized microbiome reporting is needed to advance the field.

## 5. Conclusions

This scoping review synthesized data on the relationship between the microbiome and brain tumor growth, progression, and treatment response. The current evidence highlights a robust association between the microbiome and tumor development, with emerging data supporting a bidirectional relationship where the microbiome may predict treatment response and cancer therapies impact the host microbiome. Microbiome induced immunomodulation is a promising pathogenetic mechanism behind this relationship and requires further exploration. Much of the existing evidence is preclinical, underscoring the need for clinical studies to better elucidate the microbiome–brain tumor interplay. The hope is this expanding body of knowledge will yield critical insights in the near future. Future systematic reviews, including living systematic reviews, will be essential to keep pace with this evolving field.

## Figures and Tables

**Table 1:** Characteristics of studies included in the scoping review. This table outlines the characteristics of the various studies included in the review. If information was not reported it is marked as N/A. TMZ = temozolomide.

Primary Author	Year	Country	Type of Study	Type of Publication	Human Involvement (Y/N)	Mouse Involvement (Y/N)	Type of Brain Tumor	Microbiome Source	Objective
D’Alessandro G	2020	Italy	Case–Control	Article	N	Y	Glioma	Cecum	Investigate the impact of gut microbiome alterations on glioma growth
Herbreteau A	2022	France	Case–Control	Article	N	Y	Glioma	Cecum	Investigate the impact of glioma development on GI function and microbiota
De Cecco L	2022	Italy	Cohort	Abstract	Y	N	DIPG	Feces	Determine gut microbiota in DIPG before and after radiotherapy
Dees K	2021	USA	Cohort	Article	N	Y	GBM	Feces	Determine the effect of microbial communities on glioma growth and immunotherapy response in a human mouse microbiome model
Dono A	2020	USA	Cohort	Article	Y	Y	GBM and diffuse astrocytoma	Feces	Explore the role of fecal short chain fatty acids in glioma growth
Fan H	2024	China	Cohort	Article	N	Y	Glioma	Feces	Investigate the impact of *Bifidobacterium* on glioma growth
Fan Y	2022	China	Cohort	Article	N	Y	Glioma	Feces	Investigate the effects of glioma growth on gut microbiome, and impact of gut dysbiosis on glioma development
Gomez-Manzano C	2021	USA	Case–Control	Abstract	N	Y	Glioma	Feces	Evaluate the changes in gut microbiome after treatment with viroimmunotherapy
Hermida LC	2021	USA	Cohort	Abstract	N	N	Low grade glioma	Tumor	Investigate whether can use a tumor’s microbial abundances to predict clinical outcomes and drug response
Hoogendijk R	2023	Netherlands	Case–Control	Abstract	Y	N	High grade glioma	Feces	Compare the gut microbiome composition in pediatric patients with high grade brain tumors compared to healthy controls
Hou X	2023	USA	Case–Control	Article	N	Y	Glioma	Feces	Reveal the potential role of gut microbiota in glioma development and individualized efficacy of TMZ using integrated microbiomics and metabolomics analysis
Jiang H	2022	China	Case–Control	Article	Y	N	Glioma and meningioma	Feces	Investigate the correlation between gut microbiota and benign and malignant brain tumors
Jiang H	2023	China	Case–Control	Article	Y	N	Brain Metastasis	Feces	Investigate the relationship between the microbiome and its metabolites in NSCLC, included subgroup of brain metastasis
Johnson S	2021	USA	Cohort	Abstract	Y	y	Brain Metastasis	Feces and oral	Explore the role of microbiota in brain metastases development
Ju C	2024	China	Mendelian randomization	Article	N	N	GBM	N/A	Analyze the causal association between gut microbiota and glioblastoma multiforme (GBM) using Mendelian randomization
Kim D	2024	USA	Case–Control	Abstract	N	Y	Brain Metastasis and GBM	Feces	Investigate whether the gut microbiome can influence immunotherapy response for GBM
Kim H	2023	South Korea	Case–Control	Abstract	N	Y	GBM	Not reported	Evaluate how the gut microbiome influences GBM
Kim J	2023	South Korea	Case–Control	Article	N	Y	GBM	Feces	Determine the effects of short-term supplementation with a high-glucose drink (HGD) on GBM growth and the anti-tumor immune response in mice
Ladomersky E	2019	USA	Cohort	Abstract	N	Y	GBM	N/A	Explore the impact of microbiome depletion on immunotherapy efficacy
Li H	2021	China	Case–Control	Abstract	Y	Y	Brain Metastasis	Feces	Explore the impact of gut microbiome on brain metastasis development in NSCLC
Li T	2023	China	Cohort	Abstract	Y	Y	Glioma	Brain Tissue	Investigate the microbial community composition in glioma tissues and elucidate its role in glioma development
Li X	2021	China	Cohort	Article	N	Y	Glioma	Feces	Explore gut microbiome alterations during glioma growth
Li Y	2022	China	Case–Control	Article	Y	N	Benign and Malignant Brain Tumors	Feces	Examine alterations in gut microbiota in patients with brain tumors
Lu H	2021	China	Case–Control	Article	Y	N	Brain Metastasis	Sputum and Feces	Explore microbiome profiles in patients with NSCLC
McFarland B	2017	USA	Case–Control	Abstract	N	Y	Glioma	Gut	Determine if ketogenic diet is an effective treatment for glioma, and correlate this with gut microbiota changes
Melendez-Vazquez N	2024	USA	Case–Control	Article	N	Y	GBM	Feces	Assess whether gut bacterial signatures are associated with oncolytic viral therapy efficacy
Morad G	2022	USA	Case–Control	Abstract	Y	N	Primary Brain Tumor and Brain Metastasis	N/A	Identify oral and gut microbiome signatures in primary brain tumors and brain metastasis
Morad G	2021	USA	Case–Control	Abstract	Y	N	Brain Metastasis	Feces, sputum and buccal	Evaluate the impact of microbiome depletion on melanoma brain metastasis growth
Patrizz A	2020	USA	Case–Control	Article	Y	Y	Glioma	Feces	Identify gut microbiota changes in glioma growth and in response to temozolomide
Rosito M	2024	Italy	Case–Control	Article	N	Y	Glioma	N/A	Investigate the role of dysbiosis induced by the administration of non-absorbable antibiotics on mouse metabolome and on tumor microenvironment
Strong M	2016	USA	Cohort	Article	N	N	GBM	Tumor	Identify the relationship between GBM and human cytomegalovirus
Wang J	2021	China	Case–Control	Abstract	Y	Y	Brain Metastasis	Feces and sputum	Investigate the role of the microbiome on metastatic NSCLC
Wang L	2022	China	Case–Control	Article	N	Y	Glioma	Feces	Investigate the impact of probiotic strains on glioma growth
Wang S	2024	China	Mendelian Randomization	Article	N	N	GBM	N/A	Analyze the causal association between gut microbiota and glioblastoma multiforme (GBM) using Mendelian randomization
Weathers S	2022	USA	Cohort	Abstract	Y	N	GBM	Feces	Identify factors that predict response to atezolizumab, temozolomide and radiation
Wen Y	2021	China	Case–Control	Article	Y	N	Glioma	Saliva	Investigate the relationship between oral microbiota and glioma grade
Yang J	2020	South Korea	Case–Control	Article	Y	Y	Primary Brain Tumor	Serum	Determine the predictive model for brain tumors based on microbiome signatures and extracellular vesicles
Zeng C	2023	China	Mendelian Randomization	Article	N	N	GBM	N/A	Uncover the causal relationship between glioblastoma and the gut microbiome using Mendelian randomization analysis
Zhou M	2023	China	Case–Control	Abstract	Y	N	Glioma	Feces	Profile the gut microbiome and metabolome in fecal samples from healthy volunteers and compare to those with gliomas
Zhou J	2022	China	Case–Control	Article	Y	N	Glioma	Feces	Investigate gut microbiota in recurrent glioma on bevacizumab and /or temozolomide

**Table 2:** Relationship between the microbiome and brain tumor growth and development. These are the included studies that focused on the interaction between the microbiome and brain tumor growth and development. Studies that included primary brain tumors and/or brain metastasis were included. PFS = progression free survival, OS = overall survival, TMZ = temozolomide, F/B = Firmicutes (now called *Bacillota*) to Bacteroides.

Author	Year	Brain Tumor Type	Population	Study Design	Impact on Glioma Growth or Development
D’Alessandro, G	2020	Glioma	Mice	-Glioma mouse model was treated with five weeks of antibiotics	-Tumor volume increased in mice treated with oral vancomycin and gentamicin -Interruption of antibiotic treatment decreased tumor size -In antibiotic treated mice, increased abundance of *Burkholderiales* families, and decreased *Prevotellaceae*, *Rikenellacaea*, and *Helicobacteracae* families
Dono, A	2020	Glioma	Mice and humans (n = 10 glioma, n = 6 control)	-Mice implanted with glioma cells and given TMZ or placebo -Fecal sample collected from humans prior to surgical resection and analyzed	-Glioma development alters the short chain fatty acids excreted by mice -Abundance of *Bacteroides* increased after tumor development. *Akkermansia* and *Verrucomicrobia* also increased -In humans, there was no difference between bacterial alpha and beta diversity and taxa abundance in glioma vs. healthy control using 16s rRNA sequencing
Fan H	2024	Glioma	Mice (n = 26)	-Mice implanted with glioma cells received intragastric gavage of a *Bifidobacterium* mixture. T2-weighted MRI used to evaluate tumor volume, tumor and fecal samples collected to examine microbiome	-Administration of *Bifidobacterium* mixture increased median survival in mice with glioma from 42 days to 52 days, *p* < 0.05. No significant difference in hepatic or renal toxicity *-Bifidobacterium* mixture resulted in significant increase in both the Shannon and Simpson indices, *p* < 0.01 when assessing the tumor tissue microbiome -*Bifidobacterium* mixture did not affect the alpha-diversity of the gut microbiota. At the phylum level, the group receiving this mixture had higher levels of gut *Actinobacteriota* and lower levels of *Myxococcota*, *p* < 0.05
Herbreteau, A	2022	Glioma	Mice	-Mice injected with glioma cells and given antibiotics daily -Cecum harvested on day 16 and contents analyzed	-Concentration of bacterial metabolites (short-chain fatty acids) was reduced in the cecum of glioma mice -Antibiotic treatment did not change tumor size, but changed frequency of myeloid cells in tumor environment
Hoogendijk R	2023	High grade glioma	Humans (n = 33 pediatric high-grade glioma, n = 26 controls)	-Prospectively collected fecal samples from patients at diagnosis and analyzed the microbial composition	-Comparable alpha-diversity between the groups (Shannon-index *p* = 0.45), but significant beta-diversity (Permanova test *p* = 0.02)
Hou X	2023	Glioma	Mice	-Implanted glioma cells into mice and gave one group TMZ (50 mg/kg). Fecal and tumor tissue samples collected -Broad spectrum antibiotics were given to part of the TMZ group to confirm the role of the gut microbiome on TMZ sensitivity	-Gut bacteria composition significantly changed during both glioma development and TMZ treatment -Alpha diversity indexes did not significantly change during glioma development; however, beta-diversity was different between control and glioma mice, suggesting that dysbiosis is induced by glioma development -*Bacteroides* was the most dominant phylum in the glioma group, versus *Bacillota* in the control group
Fan, Y	2022	Glioma	Mice	-Mice were injected with glioma cells and then treated with antibiotics or not. A third group was randomized to fecal transplant or not	-Abundance of *Bacteroidia* and *Actinobacteria* decreased, and *Bacillota (formerly Firmicutes)* increased during glioma growth with resultant increased F/B ratio -Tumor growth significantly worsened in mice treated with antibiotics versus those not
Jiang, H	2022	Meningioma and Malignant Glioma	Humans (n = 32 meningioma, n = 27 glioma, n = 41 control)	-Collected fecal specimen of patients with newly diagnosed brain tumor within 6 h of admission -Compared to healthy controls	-In the meningioma group, most common gut microbes at phylum level: *Bacteroidetes, Bacillota*, *Proteobacteria*, *Actinobacteria*, *Fusobacteria*, and *Verrucomicrobiota* -In the glioma group, most common at phylum level: *Bacteroidetes*, *Bacillota*, *Proteobacteria*, *Fusobacteria*, *Verrucomicrobiota*, *Actinobacteria* -In the healthy control group, most common at phylum level included *Firmictutes*, *Bacteroidetes*, *Proteobacteria*, *Actinobacteria*, *Verrucomicrobiota*, *Fusobacteria* -Alpha diversity indices all reduced in brain tumor groups compared to control
Jiang H	2023	Brain Metastasis	Humans (n = 40 brain metastasis, n = 35 healthy controls)	-Collected fecal samples from healthy controls and treatment naïve patients with metastatic NSCLC with brain metastasis, and characterized the intestinal microbiome and fecal short-chain fatty acid (SCFA) levels, which are produced by the gut microbiota	-Alpha diversity less abundant in patients with NSCLC with brain metastasis compared to healthy controls -Significant difference in beta diversity between groups -Increase in pathogens in *Fusobacteria* and *Proteobacteria* and a decrease in SCFA-producing bacteria in *Bacillota* and *Actinobacteria*, particularly in the BM stage
Johnson, S	2021	Brain metastasis	Humans (number not reported) and Mice	-Depleted gut microbiota in mice and injected melanoma cells intracranially -Evaluated tumor growth, and gut and oral microbial signatures	-Distinct enrichment pattern of bacterial and viral taxa within gut and oral microbiota in brain metastasis patients -Gut microbiome depletion decreased tumor growth in mice
Ju C	2024	GBM	Human dataset	-Took two datasets from genome-wide association studies and utilized mendelian randomization to determine causal relationship between gut microbiota and GBM	-Family *Ruminococcaceae* was shown to be protective against glioblastoma -An increase in the two microbial families, *Bacteroidaceae* and *Peptococcaceae* were associated with a high risk of GBM development -An increase in four microbial genera, *Eubacterium*, *Actinomyces*, *Bacteroides*, and *Ruminiclostridium6*, were found to be associated with an increased risk of GBM
Kim, H	2023	GBM	Mice	-Examined gut microbiome composition in mice with GBM	-Observed a distinct change in gut microbial composition and metabolism during GBM progression -Found that tryptophan levels significantly reduced in GBM mice, and thus supplemented diet with tryptophan and found it improved survival in a commensal microbiota-dependent manner
Li, H	2021	Brain Metastasis	Humans (n = 60 brain metastasis, n = 25 without brain metastasis) and Mice	-Collected fecal samples from patients with NSCLC with or without brain metastasis -Utilized mouse glioma model and gave one group antibiotics	-No differences in microbial diversity between samples from patients with and without brain metastases -*Blautia* genus decreased in brain metastasis -Antibiotics reduced tumor burden in mice, and was associated with increased microglia cells
Li, T	2023	Glioma	Humans (n = 50) and mice	-Took tumor tissue and adjacent normal brain tissue from glioma patients and performed microbial profiling, transcriptome sequencing, and metabolomics -Then, took six genera of bacteria found to be enriched in glioma tissue and developed an animal model to validate their impact on glioma growth	-Found six genera were enriched in tumor tissue (*Fusobacterium*, *Longibaculum*, *Intestinimonas*, *Pasteurella*, *Limosilactobacillus*, and *Arthrobacter*) -In vivo and in vitro models of glioma demonstrated that *Fusobacterium nucleatum* promotes glioma proliferation and upregulates CCL2, CXCL1, and CXCL2
Li, X	2021	Glioma	Mice	-Implanted mice with glioma cells and treated with TMZ for five days vs. placebo -Collected fecal samples day 0, 7, 14, 28 post-tumor implant	-Abundance of *Lactobacillus* decreased, and *Intestinimonas* and *Anaerotruncus* increased in mice with glioma compared to naïve before glioma cell implantation -With 7 days of TMZ treatment, at the phylum level there was increase in *Verrucomicrobia* compared to control; however, no change in alpha-diversity between groups -At the end of TMZ treatment, there was a significant difference in 20 genera between groups
Li, Y	2022	Benign and Malignant Primary Brain Tumors	Humans (n = 101 brain tumors, n = 57 healthy control)	-Included patients with benign and malignant brain tumors and healthy controls, collected fecal samples 2 h post hospital admission	-Gut microbial alpha diversity lower in brain tumor patients -Brain tumor cohort had higher levels of *Bacteroidetes*, *Fusobacteria*, and *Proteobacteria*, and *lower Bacillota* and *Actinobacteria* -Decreased F/B ratio in brain tumor group -*Fusobacterium*, *Enterobacteriaceae* and *Escherichia/Shigella* were all overexpressed in brain tumor patients -*Parasutterella*, *Bifidobacterium* and *Lachnospira* all enriched in healthy controls
Lu, H	2021	Brain Metastasis	Humans (n = 87 NSCLC, n = 34 healthy controls)	-Collected sputum and stool samples from patients with NSCLC and healthy controls	-Alpha-diversity in the gut lower in healthy control group -Different beta-diversities in sputum but not in feces in those with brain metastasis vs. NSCLC without brain metastasis -*Pseudomonas* was abundant in sputum and feces of brain metastasis patients, and not detectable in those without
Melendez-Vazquez, N	2024	GBM	Mice	-Created a GBM bearing mouse line and treated with either oral indoximod, Delta-24-RGDOX by intratumoral injection, or placebo. Also compared to tumor free (naïve) mouse group. -Collected fecal samples and examined bacterial composition and diversity associated with therapy	-Prominent differences in bacterial diversity between naïve and PBS treated mice, highlighting that tumor presence is associated with gut biota changes
Morad, G	2022	Primary and Metastatic Brain Tumors	Humans (n = 10 per tumor type)	-Collected stool, saliva, and buccal samples from patients with primary or metastatic brain tumors	-Distinct bacterial and viral signatures enriched in metastatic brain tumors compared to primary brain tumors
Patrizz, A	2020	Glioma	Mice and humans (n = 53)	-Implanted glioma cells into mice -Collected fecal samples prior to tumor implant, before and after TMZ or placebo -Collected stool samples from patients with glioma at diagnosis, before and after chemoradiation	-A significant decrease in the *Bacillota* to *Bacteroides* (F/B) ratio in mice suggesting dysbiosis following tumor-growth -*Bacillota* decreased and *Verrucomicrobia* phyla increased with tumor growth in mice -No difference in alpha diversity indices between controls, IDH wild-type, or IDH mutant patients at baseline -There was significant difference in F/B ratio between IDH wild-type and mutant patients compared to controls -Marked differences were observed at the phylum level in IDH-WT patients compared to controls, with increased *Bacteroidetes*, *Proteobacteria*, and *Verrucomicrobia*
Rosito M	2024	Glioma	Mice	-Treated one mouse group with oral vancomycin and gentamicin for two weeks and then transplanted glioma wells. The comparator glioma group was not treated with antibiotics.	-Found that there was a significant increase in tumor volume in antibiotic treated mice compare to mice not treated with antibiotics -Antibiotic treated mice had increased expression of CD34+ vessel like structures, suggestive of increased vasculogenesis
Strong, M	2016	GBM and LGG	Humans (n-170 GBM, n = 531 LGG)	-Utilized publicly available sequencing datasets from the Cancer Genome Atlas and whole genome datasets and normal matched blood samples -Obtained and analyzed three primary GBM tissue samples -Performed comprehensive virome assessment in both	-HPV and Hepatitis B detected in some LGG samples (4 and 1, respectively) -Concluded that likely no association between viruses and GBM development
Wang, J	2021	Brain metastasis	Mice and humans (n-87 NSCLC, n = 34 healthy volunteers)	-Treated mice with antibiotics for 2 weeks before injecting with lung cancer cells -Collected sputum and fecal samples from patients with NSCLC and healthy controls	-*Pseudomonas aeruginosa* was associated with brain metastasis
Wang, L	2022	Glioma	Mice	-Mice were treated with different probiotic cocktails and then injected with glioma cells. Fecal and tissue samples collected	-Tumor growth declined in mice treated with *Bifidobacterium lactus* and *Lactiplantibacillus plantarum* -This impact was found to be through the PI3K/AKT pathway
Wang S	2024	GBM	Human dataset	-Took a human gut microbiota dataset and utilized mendelian randomization to analyze the causal association between gut microbiota and GBM	-Found that the family *Peptostreptococcaceae* and genus *Eubacterium brachy* group were associated with increased risk of GBM -Family *Ruminococcaceae*, genus. *Anaerostipes*, genus. *Faecalibacterium*, genus. *LachnospiraceaeUCG004*, genus. *Phascolartobacterium*, genus. *Prevotella7*, and genus. *Streptococcus* were associated with reduced risk of GBM -Found family *Ruminococcaceae* to be protective against GBM (OR = 0.04, 95% CI 0.01–0.19)
Wen, Y	2021	HGG and LGG	Humans (n = 23 HGG, n = 12 LGG, n = 24 controls)	-Collected saliva samples from patients with HGG, LGG and healthy controls	-HGG was associated with a shift in oral microbiota beta-diversity -Genera *Capnocythophaga* and *Leptotrichia* were associated with glioma grade -Genera *Bergeyella* and *Capnocytophaga* were correlated with IDH1 mutation in glioma -The oral microbial features (*Capnocythophaga Porphyromonas*, *Haemophilus Leptotrichia*, and *TM7x*) discriminated HGG from LGG
Yang, J	2020	Glioma and Metastatic Brain Tumor	Mice and humans (n = 152 brain tumor patients, n = 198 control)	-Extracted extracellular (EV) vesicles that are produced by bacteria from serum of brain tumor patients and healthy controls, and glioma mouse model tissues -Then, created diagnostic models using the EV data	-Alpha diversity and beta diversity of the serum EV microbiome differed between brain tumor group and healthy controls -*Bacillota* abundance was lower in control group, and *Actinobacteria* and *Proteobacteria* were higher
Zeng C	2023	GBM	Human dataset	-Took a human gut microbiota dataset and utilized mendelian randomization to analyze the causal association between gut microbiota and GBM	*-Eubacterium brachy group*, *Eubacterium ruminantium group*, *Prevotella7*, and *Peptostreptococcaceae* were confirmed in two Mendelian Randomization methods to exhibit causality with GBM -*Ruminococcaceae* demonstrated causality with GBM in three distinct Mendelian Randomization methods
Zhou M	2023	Glioma	Humans (n = 78 glioma patients, n = 37 healthy controls)	-Collected fecal samples from healthy controls and patients with glioma, and profiled the gut microbiome and metabolome	-Found 56 discriminatory operational taxonomic units and 144 metabolites in samples from patients with gliomas compared to control -Patients with higher proportion of fecal * Faecalibacterium * had significantly better median PFS (495 d vs. 281 d, * p * = 0.005) and median OS (604 d vs. 395 d, * p * = 0.044)

**Table 3:** Relationship between the microbiome and response to therapy. These were the included studies that examined the impact of the microbiome on response to systemic and radiotherapies, and conversely, the impact of those therapies on the microbiome. PFS = progression free survival, OTU (operational taxonomic units), F/B = Firmicutes (now called *Bacillota*) to Bacteroides, TMZ = temozolomide, Bev = bevacizumab.

Author	Year	Brain Tumor Type	Population	Study Design	Treatments Included	Impact on Treatment
Dees K	2021	Glioma	Mice	-Generated five colonies of mice transplanted with different gut microbiomes transplanted from human donors; injected them with glioma cells -Mice randomized to receipt of anti-PD-1, TMZ, or placebo	Anti-PD-1 or TMZ	-Anti-PD-1 improved survival in 2/5 strains of mice -Increased CD8+/Treg ratio in responder strains -Responder mice colonies had high levels of *Bacteroides cellulosilyticus* and most similar gut microbiomes -TMZ improved survival in all five strains
De Cecco L	2022	DIPG	Humans (n = 18)	-Collected fecal samples in children with DIPG before and after RT	RT	-*Flavobacteriaceae* and *Bacillales* associated with increased risk of disease progression -*Synergistaceae* was associated with decreased risk of progression -F/B ratio changed with RT, however the direction in which is not reported
Dono A	2020	Glioma	Mice and Humans (n = 15)	-Mice implanted with glioma cells and given TMZ or placebo -Fecal sample collected from humans prior to surgical resection and analyzed	TMZ vs. placebo	-TMZ increased three fecal metabolites (acetylcholine, 3-methyl valerate, caproate) and decreased histamine -No difference in gut alpha diversity OTU or Shannon Diversity Index before and after TMZ -TMZ diminished the microbiome changes seen with glioma growth
Gomez-Manzano C	2021	Glioma	Mice	-Mice injected with Delta-24-RGDOX or saline Used immunocompetent and CD4+ depleted mouse strains	Delta-24-RGDOX vs. placebo	-Mice with intact T cells treated with therapy had increased Actinobacteria compared to control mice -*Bifidobacterium* and *Lactobacillus* associated with better response to therapy -No difference in fungal species, which was dominated by *Ascomycota*
Hou X	2023	Glioma	Mice	-Implanted glioma cells into mice and gave one group TMZ (50 mg/kg). Fecal and tumor tissue samples collected -Broad spectrum antibiotics were given to part of the TMZ group to confirm the role of the gut microbiome on TMZ sensitivity	TMZ	-Gut bacteria composition significantly changed during both glioma development and TMZ treatment -Alpha diversity indexes did not significantly change during glioma development; however, beta-diversity was different between control and glioma mice, suggesting that dysbiosis is induced by glioma development. -*Bacteroides* was the most dominant phylum in the glioma group, versus *Bacillota* in the control group -Treatment with TMZ reversed the gut microbiome dysbiosis that was induced by glioma. It let to significantly increased alpha diversity indexes (Shannon and Simpson), suggesting elevated microbial community richness and diversity. -At the phylum level, TMZ treated mice were dominated by *Firmictutes*, versus *Bacteroides* for the non-TMZ group
Kim D	2024	Brain Metastasis and GBM	Mice	-Obtained fecal samples from GBM and melanoma brain metastases (MBM) patients prior to any treatment and transplanted these microbiome samples into mice. These mice were then injected with glioma cells	Anti-PD-1	-Two strains of mice (one MBM and one GBM strain) were resistant to anti-PD-1 whereas the other two strains responded and showed prolonged survival compared to control -Ongoing microbiome sequencing underway to identify microbiome profiles of each strain
Kim J	2023	GBM	Mice	-Developed a GBM mouse model and provided them with a high glucose drink (HGD) versus a control of normal drinking water. Treated with anti-PD-1, and then tried to augment response with administration of probiotics	Anti-PD-1	-Found no effect of anti-PD1 on their mouse model. Tried to augment this with probiotic administration and found none had a significant impact on survival
Ladomersky E	2019	GBM	Mice	-Engrafted mice with glioma cells -Fed mice tryptophan and gave one set antibiotics to deplete microbiome	RT and/or anti-PD-1	-Depletion of gut microbiome had no effect on therapeutic efficacy
Li X	2021	Glioma	Mice	-Implanted mice with glioma cells and treated with TMZ for five days -Collected fecal samples day 0, 7,14,28 post tumor implant	TMZ vs. placebo	-Abundance of *Lactobacillus* decreased, and *Intestinimonas* and *Anaerotruncus* increased in mice with glioma compared to naïve -TMZ increased abundance of *Verrucomicrobia* at the phyla level, and of *Akkermansia*, *Bifidobacterium*, *Coprobacillus* and *Clostridium_XVIII* at the genus level in the first 7 days -At 21 days, TMZ increased abundance of *Anaerotruncus* genus as well as overall community diversity
Melendez-Vazquez, N	2024	GBM	Mice	-Created a GBM bearing mouse line and treated with either oral indoximod, Delta-24-RGDOX by intratumoral injection, or placebo. Also compared to tumor free (naïve) mouse group -Collected fecal samples and examined bacterial composition and diversity associated with therapy	Delta-24-RGDOX (a novel oncolytic adenovirus)	-Increased survival in those treated with Delta-24-RGDOX compared to indoximod or PBS (168 d vs. 53.4 d vs. 51.3 d, respectively) -Distinct gut microbiome composition in those treated with Delta-24-RGDOX versus placebo (*p* = 0.007) -Similar alpha-diversity levels between indoximod, Delta-24-RDGOX, and naïve group, suggesting that both immunomodulators modify diversity to be similar to the healthy naïve group -Indoximod and Delta-24-RGDOX treated mice had reduced F/B ratio, similar to naïve mice. Placebo treated mice with brain tumor had higher F/B ratio. This suggests that the immunomodulators contribute to reversal of tumor associated dysbiosis
Patrizz A	2020	Glioma	Mice and humans (n = 53)	-Implanted glioma cells into mice -Collected fecal samples prior to tumor implant, before and after TMZ or placebo -Collected stool samples from patients with glioma at diagnosis, before and after chemoradiation	TMZ	-TMZ prevented the decrease in F/B ratio therefore preventing glioma induced dysbiosis -F/B ratio differed between IDH-WT AND IDH-Mut patients. WT had increased *Bacteroidetes*, *Proteobacteria*, and *Verrucomicrobia* -No difference in F/B ratio in humans post treatment with TMZ compared to before treatment with TMZ
Weathers S	2022	GBM	Humans (n = 60)	-Collected baseline fecal samples in patients newly diagnosed with GBM -Assessed OS and PFS	Concurrent radiation, atezolizumab and TMZ, followed by atezolizumab and TMZ	-One distinct taxa (*Ruminococcus* species) were associated with OS -One distinct taxa (*Eubacterium* species) were associated with response to treatment
Zhou J	2022	WHO grade III/IV glioma	Humans (n = 29, 15 TMZ + Bev, 14 TMZ)	-Randomized patients to Bevacizumab + TMZ vs. TMZ alone, and collected fecal samples post treatment	Bevacizumab + TMZ (Group 1) versus TMZ alone (Group 2)	-Significant difference in beta-diversity of microbiome between groups -Group 1 had higher levels of *Actinobacteria*, *Bacillota* and *Bacteroidetes*

**Table 4:** Dietary changes and their impact on the gut microbiome and brain tumor outcomes. These were the included studies that examined various dietary changes and their impact on the microbiome and brain tumor outcomes.

Author	Year	Brain Tumor Type	Population	Study Design	Impact on Glioma Growth or Development
Kim H	2023	GBM	Mice	-Developed a GBM mouse model and looked at gut microbial composition and metabolism. Then, supplemented tryptophan to diet of these mice	-Found that dietary supplementation of tryptophan to GBM mice improved survival in a commensal microbiota-dependent manner
Kim J	2023	GBM	Mice	-Developed a GBM mouse model and provided them with a high glucose drink (HGD) versus a control of normal drinking water	-Found that starting HGD supplementation 5 weeks before tumor cell inoculation improved survival, versus starting after inoculation did not change outcomes. When they tried this in a germ-free mouse model, there was no difference in survival
McFarland B	2017	Glioma	Mice	-Utilized a glioma mouse model and fed them a ketogenic versus normal diet, and then compared gut microbiome between the two as well as glioma related outcomes	-Mice fed ketogenic diet had slightly increased survival compared to mice fed a normal diet -Some long-term survivors on the ketogenic diet had significant increase in gut *Faecalibaculum rodentium*

## Appendix A

Microbiome—Glioma

Final Strategies

2023 January 6

Ovid Multifile

Database: Embase Classic+Embase <1947 to 5 January 2023>, Ovid MEDLINE(R) ALL <1946 to 5 January 2023>, EBM Reviews—Cochrane Central Register of Controlled Trials <December 2022>, EBM Reviews—Cochrane Database of Systematic Reviews <2005 to 4 January 2023>

Search Strategy:

--------------------------------------------------------------------------------

1 exp Brain Neoplasms/(388260)

2 ((brain? or cerebral* or cerebell* or cerebri or cerebrum or intracerebral* or intra-cerebral* or intracran* or intra-cran* or midline or subtentorial or sub-tentorial or supratentorial or supra-tentorial) adj3 (cancer* or malignan* or neoplasm? or tumo?r?)).tw,kw,kf. (219330)

3 (cerebroma? or encephalophyma?).tw,kw,kf. (20)

4 exp Glioma/(269352)

5 exp Astrocytoma/(149378)

6 Gliosarcoma/(2573)

7 Oligodendroglioma/(14212)

8 glioma?.tw,kw,kf. (168568)

9 ((glia or glial) adj3 (malignan* or neoplasm? or tumo?r?)).tw,kw,kf. (9004)

10 (astrocytoma? or astro-cytoma? or astroglioma? or astro-glioma? or oligoastrocytoma? or oligo-astrocytoma? or oligoastro-cytoma? or oligo-astro-cytoma?).tw,kw,kf. (45870)

11 (glioblastoma? or glio-blastoma? or glyoblastoma? or glyo-blastoma? or gliosarcoma? or glio-sarcoma? or glyosarcoma? or glyo-sarcoma?).tw,kw,kf. (124971)

12 (oligodendroglioma? or oligodendro-glioma? or oligo-dendroglioma? or oligo-dendro-glioma? or olegodendrocytoma? or olegodendro-cytoma? or olego-dendrocytoma? or olego-dendro-cytoma? or oligodendrocytoma? or oligodendro-cytoma? or oligo-dendrocytoma? or oligo-dendro-cytoma? or oligo-dendrocytes#s? or oligodendro-cytos#s? or oligo-dendro-cytes#s? or oligo-dendro-cytos#s? or oligodendroblastoma? or oligodendro-blastoma? or oligo-dendroblastoma? or oligo-dendro-blastoma?).tw,kw,kf. (12200)

13 ((brain? or cerebral* or cerebell* or cerebri or cerebrum or intracerebral* or intra-cerebral* or intracran* or intra-cran* or midline or subtentorial or sub-tentorial or supratentorial or supra-tentorial) adj3 (metasta* or meta-sta* or micrometasta* or micro-metasta*)).tw,kw,kf. (64789)

14 or/1-13 [GLIOMA ETC] (663450)

15 Microbiota/(57536)

16 Microbiome/(60652)

17 (microbiome? or micro* biome? or microbiota? or micro-biota? or bacterial biome? or bacteriobiome? or bacterio-biome? or bacteriome? or fung* biome? or myco-biome? or phagome? or viral biome? or virus$2 biome? or viralbiome? or virobiome? or virobiota? or virome?).tw,kw,kf. (254030)

18 Gastrointestinal Microbiome/(111393)

19 ((alimentary or bowel? or digesti* or enteric* or gastric* or gut or GI or intestin* or gastrointestin* or gastro-intestin* or caecal or cecal or cecum or colon or colon? or colonic or duodenum or faecal or fecal or feces or ileum or jejunum or stomach or stool? or anal or anally or anus$2 or rectal$2 or rectum?) adj3 (bacteria? or bacterium or flora? or microb* or micro-b* or microflora? or micro-flora? or microbe? or microorganism? or micro-organism?)).tw,kw,kf. (260871)

20 ((mouth? or oral or throat? or dental or tooth or teeth) adj3 (bacteria? or bacterium or flora? or microb* or micro-b* or microflora? or micro-flora? or microbe? or microorganism? or micro-organism?)).tw,kw,kf. (37515)

21 Dysbiosis/(19807)

22 (dysbios#s or dysbiotic* or dys-bios#s or dys-biotic* or disbios#s or disbiotic* or dis-bios#s or dis-biotic* or dysbacterios* or dys-bacterios* or disbacterios* or dis-bacterios* or dyssymbio* or dys-symbio* or dissymbio* or dis-symbio*).tw,kw,kf. (39137)

23 Brain-Gut Axis/(1672)

24 (brain adj2 gut adj3 (ax#s or crosstalk* or cross-talk* or interplay* or inter-play or interact* or inter-act*)).tw,kw,kf. (13317)

25 or/15-24 [MICROBIOME/MICROBIOTA] (429110)

26 14 and 25 [GLIOMA ETC—MICROBIOME/MICROBIOTA] (474)

27 26 use medall [**MEDLINE RECORDS**] (111)

28 exp brain cancer/(226817)

29 ((brain? or cerebral* or cerebell* or cerebri or cerebrum or intracerebral* or intra-cerebral* or intracran* or intra-cran* or midline or subtentorial or sub-tentorial or supratentorial or supra-tentorial) adj3 (cancer* or malignan* or neoplasm? or tumo?r?)).tw,kw,kf. (219330)

30 (cerebroma? or encephalophyma?).tw,kw,kf. (20)

31 exp glioma/(269352)

32 exp astrocytoma/(149378)

33 gliosarcoma/(2573)

34 oligodendroglioma/(14212)

35 glioma?.tw,kw,kf. (168568)

36 ((glia or glial) adj3 (malignan* or neoplasm? or tumo?r? or metasta* or meta-sta* or micrometasta* or micro-metasta*)).tw,kw,kf. (9057)

37 (astrocytoma? or astro-cytoma? or astroglioma? or astro-glioma? or oligoastrocytoma? or oligo-astrocytoma? or oligoastro-cytoma? or oligo-astro-cytoma?).tw,kw,kf. (45870)

38 (glioblastoma? or glio-blastoma? or glyoblastoma? or glyo-blastoma? or gliosarcoma? or glio-sarcoma? or glyosarcoma? or glyo-sarcoma?).tw,kw,kf. (124971)

39 (oligodendroglioma? or oligodendro-glioma? or oligo-dendroglioma? or oligo-dendro-glioma? or olegodendrocytoma? or olegodendro-cytoma? or olego-dendrocytoma? or olego-dendro-cytoma? or oligodendrocytoma? or oligodendro-cytoma? or oligo-dendrocytoma? or oligo-dendro-cytoma? or oligo-dendrocytes#s? or oligodendro-cytos#s? or oligo-dendro-cytes#s? or oligo-dendro-cytos#s? or oligodendroblastoma? or oligodendro-blastoma? or oligo-dendroblastoma? or oligo-dendro-blastoma?).tw,kw,kf. (12200)

40 brain metastasis/(41661)

41 ((brain? or cerebral* or cerebell* or cerebri or cerebrum or intracerebral* or intra-cerebral* or intracran* or intra-cran* or midline or subtentorial or sub-tentorial or supratentorial or supra-tentorial) adj3 (metasta* or meta-sta* or micrometasta* or micro-metasta*)).tw,kw,kf. (64789)

42 or/28-41 [GLIOMA ETC] (581611)

43 microflora/(29823)

44 microbiome/(60652)

45 (microbiome? or micro* biome? or microbiota? or micro-biota? or bacterial biome? or bacteriobiome? or bacterio-biome? or bacteriome? or fung* biome? or myco-biome? or phagome? or viral biome? or virus$2 biome? or viralbiome? or virobiome? or virobiota? or virome?).tw,kw,kf. (254030)

46 exp intestine flora/(90184)

47 ((alimentary or bowel? or digesti* or enteric* or gastric* or gut or GI or intestin* or gastrointestin* or gastro-intestin* or caecal or cecal or cecum or colon or colon? or colonic or duodenum or faecal or fecal or feces or ileum or jejunum or stomach or stool? or anal or anally or anus$2 or rectal$2 or rectum?) adj3 (bacteria? or bacterium or flora? or microb* or micro-b* or microflora? or micro-flora? or microbe? or microorganism? or micro-organism?)).tw,kw,kf. (260871)

48 exp mouth flora/(10600)

49 ((mouth? or oral or throat? or dental or tooth or teeth) adj3 (bacteria? or bacterium or flora? or microb* or micro-b* or microflora? or micro-flora? or microbe? or microorganism? or micro-organism?)).tw,kw,kf. (37515)

50 dysbiosis/(19807)

51 (dysbios#s or dysbiotic* or dys-bios#s or dys-biotic* or disbios#s or disbiotic* or dis-bios#s or dis-biotic* or dysbacterios* or dys-bacterios* or disbacterios* or dis-bacterios* or dyssymbio* or dys-symbio* or dissymbio* or dis-symbio*).tw,kw,kf. (39137)

52 brain-gut axis/(1672)

53 (brain adj2 gut adj3 (ax#s or crosstalk* or cross-talk* or interplay* or inter-play or interact* or inter-act*)).tw,kw,kf. (13317)

54 or/43-53 [MICROBIOME/MICROBIOTA] (435532)

55 42 and 54 [GLIOMA ETC—MICROBIOME/MICROBIOTA] (451)

56 55 use emczd [**EMBASE RECORDS**] (322)

57 exp Brain Neoplasms/(388260)

58 ((brain? or cerebral* or cerebell* or cerebri or cerebrum or intracerebral* or intra-cerebral* or intracran* or intra-cran* or midline or subtentorial or sub-tentorial or supratentorial or supra-tentorial) adj3 (cancer* or malignan* or neoplasm? or tumo?r?)).ti,ab,kw. (205138)

59 (cerebroma? or encephalophyma?).ti,ab,kw. (20)

60 exp Glioma/(269352)

61 exp Astrocytoma/(149378)

62 Gliosarcoma/(2573)

63 Oligodendroglioma/(14212)

64 glioma?.ti,ab,kw. (166796)

65 ((glia or glial) adj3 (malignan* or neoplasm? or tumo?r?)).ti,ab,kw. (8854)

66 (astrocytoma? or astro-cytoma? or astroglioma? or astro-glioma? or oligoastrocytoma? or oligo-astrocytoma? or oligoastro-cytoma? or oligo-astro-cytoma?).ti,ab,kw. (45314)

67 (glioblastoma? or glio-blastoma? or glyoblastoma? or glyo-blastoma? or gliosarcoma? or glio-sarcoma? or glyosarcoma? or glyo-sarcoma?).ti,ab,kw. (123886)

68 (oligodendroglioma? or oligodendro-glioma? or oligo-dendroglioma? or oligo-dendro-glioma? or olegodendrocytoma? or olegodendro-cytoma? or olego-dendrocytoma? or olego-dendro-cytoma? or oligodendrocytoma? or oligodendro-cytoma? or oligo-dendrocytoma? or oligo-dendro-cytoma? or oligo-dendrocytes#s? or oligodendro-cytos#s? or oligo-dendro-cytes#s? or oligo-dendro-cytos#s? or oligodendroblastoma? or oligodendro-blastoma? or oligo-dendroblastoma? or oligo-dendro-blastoma?).ti,ab,kw. (12146)

69 ((brain? or cerebral* or cerebell* or cerebri or cerebrum or intracerebral* or intra-cerebral* or intracran* or intra-cran* or midline or subtentorial or sub-tentorial or supratentorial or supra-tentorial) adj3 (metasta* or meta-sta* or micrometasta* or micro-metasta*)).ti,ab,kw. (63880)

70 or/57-69 [GLIOMA ETC] (659880)

71 Microbiota/(57536)

72 Microbiome/(60652)

73 (microbiome? or micro* biome? or microbiota? or micro-biota? or bacterial biome? or bacteriobiome? or bacterio-biome? or bacteriome? or fung* biome? or myco-biome? or phagome? or viral biome? or virus$2 biome? or viralbiome? or virobiome? or virobiota? or virome?).ti,ab,kw. (248353)

74 Gastrointestinal Microbiome/(111393)

75 ((alimentary or bowel? or digesti* or enteric* or gastric* or gut or GI or intestin* or gastrointestin* or gastro-intestin* or caecal or cecal or cecum or colon or colon? or colonic or duodenum or faecal or fecal or feces or ileum or jejunum or stomach or stool? or anal or anally or anus$2 or rectal$2 or rectum?) adj3 (bacteria? or bacterium or flora? or microb* or micro-b* or microflora? or micro-flora? or microbe? or microorganism? or micro-organism?)).ti,ab,kw. (255218)

76 ((mouth? or oral or throat? or dental or tooth or teeth) adj3 (bacteria? or bacterium or flora? or microb* or micro-b* or microflora? or micro-flora? or microbe? or microorganism? or micro-organism?)).ti,ab,kw. (36459)

77 Dysbiosis/(19807)

78 (dysbios#s or dysbiotic* or dys-bios#s or dys-biotic* or disbios#s or disbiotic* or dis-bios#s or dis-biotic* or dysbacterios* or dys-bacterios* or disbacterios* or dis-bacterios* or dyssymbio* or dys-symbio* or dissymbio* or dis-symbio*).ti,ab,kw. (38647)

79 Brain-Gut Axis/(1672)

80 (brain adj2 gut adj3 (ax#s or crosstalk* or cross-talk* or interplay* or inter-play or interact* or inter-act*)).ti,ab,kw. (11291)

81 or/71-80 [MICROBIOME/MICROBIOTA] (422540)

82 70 and 81 [GLIOMA ETC—MICROBIOME/MICROBIOTA] (469)

83 82 use coch,cctr [**COCHRANE RECORDS**] (16)

84 27 or 56 or 83 [**ALL DATABASES**] (449)

85 remove duplicates from 84 (356) [**TOTAL UNIQUE RECORDS**]

86 85 use medall [MEDLINE UNIQUE RECORDS] (110)

87 85 use emczd [EMBASE UNIQUE RECORDS] (233)

88 85 use cctr [CENTRAL UNIQUE RECORDS] (13)

89 85 use coch [CDSR UNIQUE RECORDS] (0)

***************************

Web of Science (Core Databases)

Set #	Search Query	Results
17	#16 AND #9	135
16	#12 OR #11 OR #13 OR #14 OR #15	222447
15	(“brain-gut” or “gut-brain”) NEAR/3 (axis or axes or crosstalk* or cross-talk* or interplay* or inter-play or interact* or inter-act*) (Topic)	6002
14	dysbiosis or dysbioses or dysbiotic* or “dys-biosis” or “dys-bioses” or “dys-biotic” or “dys-biotics” or disbiosis or disbioses or disbiotic* or “dis-biosis” or “dis-bioses” or “dis-biotic” or “dis-biotics” or dysbacterios* or dys-bacterios* or disbacterios* or dis-bacterios* or dyssymbio* or dys-symbio* or dissymbio* or dis-symbio* (Topic)	18291
13	(mouth or mouths or oral or throat or throats or dental or tooth or teeth) NEAR/3 (bacteria* or bacterium or flora or florae or floral or floras or microb* or micro-b* or microflora* or “micro-flora” or “micro-floras” or “micro-floral” or “micro-floras” or microbe* or microorganism* or “micro-organism” or “micro-organisms”) (Topic)	19081
12	(alimentary or bowel or bowels or digesti* or enteric* or gastric* or gut or GI or intestin* or gastrointestin* or “gastro-intestine” or “gastro-intestines” or “gastro-intestinal” or caecal or cecal or cecum or colon or colon or colons or colonic or duodenum or faecal or fecal or feces or ileum or jejunum or stomach or stool or stools or anal or anally or anus or anuses or rectal or rectally or rectum or rectums) NEAR/3 (bacteria* or bacterium or flora or florae or floral or floras or microb* or micro-b* or microflora* or “micro-flora” or “micro-floras” or “micro-floral” or “micro-floras” or microbe* or microorganism* or “micro-organism” or “micro-organisms”) (Topic)	145634
11	microbiome* or “micro-biome” or “micro-biomes” or microbiota* or “micro-biota” or “micro-biotas” or “bacterial biome” or “bacterial biomes” or bacteriobiome* or “bacterio-biome” or “bacterio-biomes” or bacteriome* or “fungal biome” or “fungal biomes” or “fungi biome” or “fungi biomes” or “fungus biome” or “fungus biomes” or “myco-biome” or “myco-biomes” or phagome* or “viral biome” or “viral biomes” or “virus biome” or “virus biomes” or viralbiome* or virobiome* or virobiota* or virome* (Topic)	152006
10	#8 OR #7 OR #6 OR #5 OR #4 OR #3 OR #2 OR #1	238544
9	#8 OR #7 OR #6 OR #5 OR #4 OR #3 OR #2 OR #1	238544
8	(brain or brains or cerebral* or cerebell* or cerebri or cerebrum or intracerebral* or intra-cerebral* or intracran* or intra-cran* or midline or subtentorial or “sub-tentorial” or supratentorial or “supra-tentorial”) NEAR/3 (metasta* or meta-sta* or micrometasta* or micro-metasta*) (Topic)	29744
7	oligodendroglioma* or “oligodendro-glioma” or “oligodendro-gliomas” or “oligo-dendroglioma” or “oligo-dendrogliomas” or “oligo-dendro-glioma” or “oligo-dendro-gliomas” or olegodendrocytoma* or “olegodendro-cytoma” or “olegodendro-cytomas” or “olego-dendrocytoma” or “olego-dendrocytomas” or “olego-dendro-cytoma” or “olego-dendro-cytomas” or oligodendrocytoma* or “oligodendro-cytoma” or “oligodendro-cytomas” or “oligo-dendrocytoma” or “oligodendro-cytomas” or “oligo-dendro-cytoma” or “oligo-dendro-cytomas” or “oligo-dendrocytesis” or “oligo-dendrocyteses” or “oligodendro-cytosis” or “oligodendro-cytoses” or “oligo-dendro-cytesis” or “oligo-dendro-cyteses” or “oligo-dendro-cytosis” or “oligo-dendro-cytoses” or oligodendroblastoma* or “oligodendro-blastoma” or “oligodendro-blastomas” or “oligo-dendroblastoma” or “oligo-dendroblastomas” or “oligo-dendro-blastoma” or “oligo-dendro-blastomas” (Topic)	5443
6	glioblastoma* or “glio-blastoma” or “glio-blastomas” or glyoblastoma* or “glyo-blastoma” or “glyo-blastomas” or gliosarcoma* or “glio-sarcoma” or “glio-sarcomas” or glyosarcoma* or “glyo-sarcoma” or “glyo-sarcomas” (Topic)	73747
5	astrocytoma* or “astro-cytoma” or “astro-cytomas” or astroglioma* or “astro-glioma” or “astro-gliomas” or oligoastrocytoma* or “oligo-astrocytoma” or “oligo-astrocytomas” or “oligoastro-cytoma” or “oligoastro-cytomas” or “oligo-astro-cytoma” or “oligo-astro-cytomas” (Topic)	21979
4	(glia or glial) NEAR/3 (malignan* or neoplasm or neoplasms or tumor or tumors or tumour or tumours) (Topic)	3954
3	glioma or gliomas (Topic)	103014
2	cerebroma* or encephalophyma* (Topic)	29
1	(brain or brains or cerebral* or cerebell* or cerebri or cerebrum or intracerebral* or intra-cerebral* or intracran* or intra-cran* or midline or subtentorial or “sub-tentorial” or supratentorial or “supra-tentorial”) NEAR/3 (cancer* or malignan* or neoplasm or neoplasms or tumor or tumors or tumour or tumours) (Topic)	108162
